# Intermittent preventive treatment: efficacy and safety of sulfadoxine-pyrimethamine and sulfadoxine-pyrimethamine plus piperaquine regimens in schoolchildren of the Democratic Republic of Congo: a study protocol for a randomized controlled trial

**DOI:** 10.1186/1745-6215-14-311

**Published:** 2013-09-24

**Authors:** Joachim Yorokpa Doua, Junior Matangila, Pascal Lutumba, Jean-Pierre Van geertruyden

**Affiliations:** 1 International Health Unit, Department of Epidemiology and Social Medicine, Faculty of Medicine and Health Sciences, University of Antwerp, Universiteitsplein 1, BE-2610, Antwerpen, Belgium; 2 Faculté de Médecine, Université de Kinshasa, B.P. 747, Kinshasa XI, République Démocratique du Congo

**Keywords:** Intermittent preventive treatment, Efficacy, Safety, Sulfadoxine-pyrimethamine resistance, Schoolchildren, Malaria, Anaemia, Piperaquine, Randomised controlled trial

## Abstract

**Background:**

In malaria endemic areas, schoolchildren usually have asymptomatic malaria infections and consequently remain untreated. Therefore, intermittent preventive treatment with sulfadoxine-pyrimethamine in schoolchildren would be a plausible strategy in malaria stable transmission areas to prevent anaemia and malnutrition. However, in contrast to infancy and pregnancy, antimalaria intermittent preventive treatment in children has been barely investigated. As the implementation of intermittent preventive treatment may be challenged by sulfadoxine-pyrimethamine resistance, sulfadoxine-pyrimethamine combined with piperaquine may be a better alternative than sulfadoxine-pyrimethamine monotherapy. A clinical trial is being conducted to assess the efficacy and safety of intermittent preventive treatments versus controls in Democratic Republic of Congo (DRCongo) schoolchildren and their impact on sulfadoxine-pyrimethamine resistance.

**Methods/Design:**

A phase IIIb, randomised, controlled trial will enroll asymptomatic schoolchildren. For interventions, sulfadoxine-pyrimethamine is compared to sulfadoxine-pyrimethamine plus piperaquine and to a control group. The two treatments are given four-monthly from baseline for a year as a single dose for sulfadoxine-pyrimethamine and two doses at 24-hour intervals for piperaquine. All participants receive praziquantel and albendazole as mass-treatment for helminthiasis at enrolment. The primary endpoint is haemoglobin concentration change at 12 months follow-up. Secondary endpoints are malaria parasite load and malaria prevalence, at baseline and at month 12. Malaria and helminthiasis incidence will be monitored throughout the study. Statistical analysis will use multilevel modelling due to repeated measurements and clustering effect of participants.

**Discussion:**

The very few studies on intermittent preventive treatment in schoolchildren in malaria stable transmission areas have contradictory results. This randomised controlled trial is unique in comparing efficacy and safety of a prophylactic combination therapy to monotherapy or a control group after 12 months follow-up. Resistance markers for sulfadoxine-pyrimethamine (including break through parasitaemias) will also be recorded. Its uniqueness lies also in the fact that we use piperaquine, a long acting antimalarial, in combination with sulfadoxine-pyrimethamine. Artemisinin derivatives have been excluded as it is part of the treatment policies in virtually all malaria endemic countries. Our findings may, therefore, contribute to the public health of youngsters who fail to thrive and grow due to multiple morbidities.

**Trial registration:**

NCT01722539; PACTR201211000449323

## Background

Malaria is a major parasitic disease in developing countries and particularly in Sub Saharan Africa (SSA). Malaria is caused by five plasmodium parasites, the most severe being *Plasmodium falciparum *[1]. More than half of the world population is at risk for malaria. Globally, 243 million clinical malaria cases and slightly more than 1 million deaths are reported annually [2]; 90% of this burden occurs in African children [3,4]. The Democratic Republic of Congo (DRCongo) is likely one of the most affected countries. The malaria transmission is perennial, malaria accounts for 86% of admissions in paediatric emergencies, and the incidence in young Congolese children is estimated at 60 to 100 million malaria attacks per year [5].

Severe anaemia is one of the leading causes of death due to malaria disease in childhood in intense and stable malaria transmission areas [6]. In endemic areas where subjects are permanently exposed to infectious mosquitos bites, severe disease develops in infants and young children. However, in children of five years of age and school-age children, malaria infection is characterised by low recurrent parasitaemia [7]. The prolonged carriage of *Plasmodium* triggers the development of acquired immunity, the premunition that somewhat protects older children and adults against clinical malaria and reduces the risk of death in school-age children [7,8]. Asymptomatic *Plasmodium* infections, if untreated, persist and maintain malaria-induced inflammation that is commonly associated with iron deficiency anaemia through impaired intestinal iron absorption and iron release from hepatocytes, and impairment of the recycling of iron derived from parasitized red blood cells phagocytosis [9]. In low income countries (LICs), more than half of the school-aged population suffers from anaemia; in SSA, approximately 85 million school-aged children are affected. Anaemia reduces their cognitive potential, retards their growth and predisposes them to other diseases [6]. Also malaria accounts about for 13% to 50% of all annual school absenteeism and impairs the educational achievement of children [10].

Intermittent preventive treatment (IPT) with sulfadoxine-pyrimethamine (SP) is a safe and efficacious control tool for malaria that combined or not with insecticidal treated nets, showed substantial protection against malaria in vulnerable populations [11-13]. IPT treats patients at long intervals, permitting drug concentrations to fall below the minimal inhibitory concentration (MIC) between treatment courses. IPT is a public health intervention that builds on clearing existing parasites (treatment effect seen in mass drug administration) to avoid clinical malaria and prevent new infections (prophylactic effect) without affecting the development of immunity. Conversely, in chemoprophylaxis, the blood concentration of the drug is maintained above the level that inhibits the growth of (pre)erythrocytic parasites [14].

IPT markedly reduces clinical malaria incidence and overall child mortality, and improves haemoglobin concentration and the nutritional status of children <5-years-old [12,15]. Additionally, IPT may decrease malaria transmission amongst the most vulnerable individuals through a post-treatment prophylactic effect [14]. IPT is delivered through antenatal consultations for pregnant (IPTp) women and through vaccination programs for infants (IPTi). A policy recommendation has been developed by the World Health Organization for IPTp and IPTi [4,16,17]. Schools have been suggested as possible delivery sites for IPT in children (IPTc). Indeed, the education sector may represent a reliable system for malaria control. IPT in schoolchildren (IPTsc) is likely the most feasible and appropriate chemoprevention in stable and endemic areas because schoolchildren are usually asymptomatic to malaria infection and are consequently untreated in practice. Therefore, if proven effective, IPTsc would be of direct benefit for the schoolchild, contribute to malaria control at school, and facilitate on a community-wide basis the implementation of other control interventions, that is, vector control, IPTi, and malaria primary diagnosis and treatment [18].

Favourable drugs for use as IPT should balance long half-life against efficacy, safety, tolerability and potentiality for cross-resistance selection [16]. Use of long-acting drugs would result in fewer intakes and higher treatment compliance. SP is an established product in the treatment of IPTp. The drug has further proven safety and tolerability in children in clinical trials. SP is slowly eliminated and provides 60-day antimalaria protection for fully sensitive *P. falciparum*.

Other long-acting drugs available are mefloquine, amodiaquine, and piperaquine. However, due to safety concerns, mefloquine might not be optimal for IPT. Amodiaquine is not suitable for IPT due to its three-day treatment regimen and is used as a first line treatment in several countries including DRCongo. Piperaquine has been extensively used for mass prophylaxis and treatment since 1978 in China and other malaria endemic countries of Asia [19]. Piperaquine has a long half-live and may be a good IPT candidate in malaria endemic countries [20].

Pyrimethamine binds to dihydrofolate reductase (DHFR) enzyme and sulfadoxine to dihydropteroate synthase (DHPS) and, thereby, the two SP components inhibit the *P. falciparum* life cycle [21]. However, SP resistance has spread globally due to *pf*DHFR and *pf*DHPS gene mutations as a consequence of an increasing proportion of individuals unnecessarily exposed to drugs [22]. Of note, antimalarials with lengthy elimination half-lives have a higher propensity of selecting for resistance due to exposure of new infecting malaria parasites to the drug when its concentration tail falls below the MIC [23].

To minimise the risk of SP resistance, SP could be combined with piperaquine (SP-PQ) as recommended by Cairns *et al.* and Gready [20,24]. Also, SP and SP-PQ, given at four-month intervals in line with the long half-lives (around 20 days) in paediatric patients, may improve treatment compliance. Nevertheless, evidence about the efficacy and safety of IPT SP and SP-PQ in the presence of SP resistance strains selection is not yet substantiated in schoolchildren. Only three clinical trials with restricted treatment arms and/or follow-up have so far been performed on IPTsc in hyper endemic areas. Further clinical trials are warranted in other settings. Through a randomised controlled trial (RCT) we will assess the efficacy and safety of IPT SP or SP-PQ regimens versus controls in schoolchildren of the DRCongo.

### Study hypothesis

Considering the facts that in our study area: (1) IPT of malaria provides substantial protection against anaemia and malaria in school children; (2) the level of SP resistance has no significant impact on the prophylactic efficacy [25]; and (3) SP-PQ is safe and as efficacious as SP, we hypothesize that antimalarial IPT with SP and SP-PQ will improve haemoglobin concentration, reduce anaemia prevalence, malaria incidence and parasitaemia, and improve nutritional status and school performance in school-aged children of DRCongo.

## Methods/Design

### Trial objectives and purpose

#### *Primary objectives*

This study primarily aims to assess the effect of antimalarial IPT on anaemia one year after initial intervention in school-aged children of stable malaria transmission and hyper endemic areas.

#### *Secondary objectives*

Secondarily, in the same study population, the purpose of the study is to:

1. Determine the prevalence of SP resistance markers at baseline and at month 12 follow-up;.

2. Compare the efficacy and safety of SP versus SP-PQ.

3. Assess the impact of antimalarial IPTsc on malaria incidence and severity.

4. Evaluate the impact of IPTsc on the nutritional status of schoolchildren.

5. Evaluate the effect of antimalarial IPTsc on school performance.

6. Identify the potential environmental and host-related predictors of malaria (re)infections.

### Study design

#### *Study area*

The study is conducted in the Mokali health area of the Biyela health zone, in Kinshasa province. The health zone of Biyela has recently been identified as highly endemic for malaria and prevalent for schistosomiasis and soil transmitted helminths (STH) (unpublished observations of Lutumba P). Biyela is urban–rural and its population is estimated at 182,421 inhabitants. Biyela has nine subzones of which two are considered rural. Three rivers cross the health zone: Mango, Mabanga and Mokali. Of the nine health areas of Biyela, Mokali is the most populous with a population estimated at 27,455 inhabitants. Biyela has one regional reference hospital that is located in the Mokali health area. The participants in the study are recruited at EP Boyambi and EP Likabo, two of the nearest primary schools of Mokali to the regional health centre. EP Boyambi and EP Likabo were built by the Catholic Church and each school has 14 teachers and 12 classes (two classes per each school year). The estimated number of schoolchildren was 650 per school at the beginning of the year.

### Patient selection and withdrawal

#### *Inclusion criteria for initial treatment*

In order to be eligible, children should satisfy all of the following criteria:

1. Male and female primary school children.

2. Anticipated local residence for the study duration.

3. Signed or thumb-printed informed consent by the parents or guardians and witnessed by an impartial witness (whenever parents/guardians are illiterate).

4. Informed consent for children 12-years-old or older.

#### *Exclusion criteria*

Participants with at least one of the following criteria are excluded:

1. Children of the sixth primary school year.

2. Participation in any other investigational drug study (antimalarial or others) during the previous 30 days.

3. Known or suspected hypersensitivity or serious adverse events (AE) to the study drugs.

4. Malaria symptoms at baseline irrespective of the severity [see Additional file 1].

5. Febrile conditions caused by diseases other than malaria at the first visit.

6. Decompensated anaemia.

7. Illness or conditions, such as hematologic, cardiac, renal, hepatic diseases, which in the judgement of the investigators would place the subject at undue risk or interfere with the results of the study, including known glucose-6-phosphate dehydrogenase deficiency and sickle cell.

8. Body weight <14 kg.

9. Children with major chronic infectious diseases (HIV, tuberculosis, and so on).

#### *Withdrawal criteria*

Patients will be excluded from further assessment if any of the following criteria are met:

1. Withdrawal of informed consent.

2. Severe AE related to the study drug.

3. Termination of the study prior to its planned end date by the sponsor, regulatory authorities, or Ethics Committee.

Every reasonable effort is made to complete a final evaluation of participants who are withdrawn from the study prior to the planned termination time period. The study staff records the reason(s) for all withdrawals from the study in the participants’ study records. At the time of withdrawal, all study procedures outlined for the End of Study visit should be completed. Any withdrawal or loss to follow-up are documented and notified to the site coordinator as soon as possible.

### Endpoints

#### *Primary endpoint*

The primary endpoint is the change in mean Hb concentration at month 12 of follow-up and anaemia prevalence one year after the initial preventive treatment.

#### *Secondary endpoints:*

1. Change in mean Hb concentration at month 4 and 8 of follow-up.

2. Prevalence of asymptomatic and clinical malaria at baseline and one year after enrolment.

3. Prevalence of *pf*DHFR and *pf*DHPS gene mutations at baseline and at month 12 follow-up.

4. Clinical (severe) malaria incidence and parasitaemia at month 4, 8 and 12.

5. Percentages of acute and severe malnutrition at month 0, 4, 8, 12 through weight/height and height/age z-scores, and skin folds.

6. School achievement at the end of follow-up and school attendance.

7. Prevalence and risk of environmental and host-related predictors for malaria (re)infections.

### Safety endpoints

Subjects are monitored throughout the study for possible development of AE. All AEs are recorded on the specific form in the Case Report Form (CRF). Any changes in relevant laboratory parameters are also assessed. For all AEs, a relative risk (RR) and also the frequency, severity and seriousness will be measured.

### Design and sample size calculation

The study is an unblinded, randomised controlled trial, enrolling asymptomatic school children, either gender. At baseline, month four, and month eight, IPT with SP or SP-PQ is given. SP is administered as a single treatment dose. However, PQ is given as two doses with a 24-hour interval. IPT is administered at four month intervals in line with the long half-lives of the drugs and for compliance. Each treatment arm will be compared to the control group of untreated participants. Due to the prevalence of STH and schistosomiasis in the study area all participants are treated with albendazole or praziquantel according to the WHO guidelines [26]. Before the study, parents or guardians and children were informed about the objectives and the design of the study during organised meetings at schools.

Within each school, each schoolchild is randomly assigned to one of the two intervention groups or the control group as follows: IPT-SP, IPT-SP-PQ and no-treatment. Each study arm consists of one-third of all individuals.

To estimate the statistical power of our studies, we used an effect size of 0.56 g/dl mean Hb level improvement by the antimalarial IPT compared to the control treatment. This effect size was based upon a previous study.

A previous study estimated the mean Hb concentration of 13.01 g/dl respectively in schoolchildren with IPT and 12.45 g/dl without [27]. The sample size of the study is all schoolchildren of two schools selected within the nearest schools to the regional hospital centre. The overall number of schoolchildren in the two schools is estimated to be 1,300 (650 in each school). By excluding 200 children of the sixth primary school year (100 per school), and accounting for 20% cancelation and 30% to 40% absenteeism, a realistic sample size of 616 individuals is reached for the complete IPT treatment schedule. Using this effect size and sample size, power estimates were performed by Monte Carlo simulation. In each run, a dataset was simulated assuming the within-subject variance and between-subject variability was 16, and putting in effect size to 1. The power was then estimated by fitting a linear mixed model to the data (Hb level versus treatment, time and time:treatment interaction) and testing the significance of the interaction term. The power is then equal to the fraction of times the interaction term was significant, using a significance level of 5%. A sample size of 616 offers 100% power to detect superiority between the control and the intervention groups. For the non-inferiority of SP versus SP-PQ, this sample size has over 91% power to detect a difference in effect less than 0.27 g/dl (<50%).

### Study procedures

#### *Baseline, enrolment and treatment allocation*

General information on each school regarding composition, water sources, sanitation, health services, and current and previous treatments are collected by questionnaire. The social, economic and demographic patterns are recorded, physical/clinical and complementary examinations are performed, and study treatments and possible co-medications are given according to the study treatment allocation and the participant’s health status (see Table 1).

**Table 1:** Follow-up chart of study participants

**Timeline in months**	**Baseline**	**4**	**8**	**12**
Informed consent	X			
Socio-economic and demographic	X			X
History (symptoms)	X	X	X	X
Examination (clinical)	X			X
Temperature	X	X	X	X
School achievement	X			X
Blood film	X	X	X	X
Hemoglobin (hemocontrol)	X	X	X	X
Thin and thick smear	X	X	X	X
Plasma sample	X			X
Filter paper PCR	X			X
Stool and urine sample	X			X
Malaria treatment^b^	X	X	X	X
Anthelminthic treatment	X	X^a^	X^a^	X
Adverse events	X	X	X	X
Concomitant medications	X	X	X	X

^a^only albendazole is given to participants. ^b^all participants will receive SP at Month 12 irrespective of the treatment allocation. In lines: we have the timelines (in months) that are displayed as baseline, month 4, month 8 and month 12. In columns are the study tasks that are performed and the parameters that are collected. X means perform this task. Blank cells mean that the specific activity will not be performed at the corresponding timeline.

#### *Follow-up visits at month 4, 8, 12*

Each study participant is followed up for 12 months. If, during the follow up, a child experiences clinical malaria or other illnesses, s/he is advised to attend the health centre for a treatment according to the national guidelines. Children absent from school at the time of the follow-up visit are seen on another day of the week for treatment and collection of three blood drops for hemocontrol and microscopy. To minimize reporting and/or recall bias, teachers in each class are encouraged to contact the site investigator when a study participant is absent or is sick (by mobile phone). Any child with a health problem is advised to consult doctors at the regional hospital of Biyela that is the health centre chosen for the study. Parents and guardians are advised to visit the health centre immediately when a child is unwell at home.

Clinical malaria cases observed during school visits are documented and referred to the Biyela regional hospital. At the end of follow-up all participants will receive one dose of SP, albendazole and praziquantel and will be passively monitored for three months [See Additional file 2].

#### *Health centre visit*

A child attending the health centre is examined and treated in line with national guidelines. All relevant medical records (history, physical and clinical examination, laboratory results, the diagnosis and the prognosis made by the clinician and the treatment given) of the patient are recorded on the CRF [see Additional file 3].

## Study drugs

### Investigational products

#### *Sulfadoxine–Pyrimethamine (SP)*

Sulfadoxine–Pyrimethamine is an established drug used for uncomplicated malaria in children and adults and for IPT in pregnant women. The safety and efficacy of SP has been widely established in infants and children in clinical trials. For the current study the Fansidar brand of SP is used. Fansidar is supplied as scored tablets, containing 500 mg sulfadoxine and 25 mg pyrimethamine unit doses. Sulfadoxine and pyrimethamine are folic acid antagonists. Sulfadoxine inhibits the activity of dihydropteroate synthase whereas pyrimethamine inhibits dihydrofolate reductase. *In vitro*, sulfadoxine and pyrimethamine are active against the asexual erythrocytic stages of *P. falciparum*. SP may also be effective against strains of *P. falciparum* resistant to chloroquine. Fansidar is approved and commercialised for the treatment of uncomplicated malaria and the prevention of malaria.

Owing to its slow elimination profile, levels of SP sufficient to inhibit parasite growth persist for 60 days after treatment, providing a period of post-treatment prophylaxis during which successful development of a blood-stage infection is prevented. After administration of one tablet, peak plasma levels for pyrimethamine (approximately 0.2 mg/L) and for sulfadoxine (approximately 60 mg/L) are reached after about four hours. The volume of distribution for sulfadoxine and pyrimethamine is 0.14 L/kg and 2.3 L/kg, respectively. Plasma protein binding is about 90% for both pyrimethamine and sulfadoxine. A relatively long elimination half-life is characteristic of both components. The mean values for plasma elimination half-lives are up to 11 days for sulfadoxine and 5 days for pyrimethamine, mainly via the kidneys [28].

Fansidar is given as a single oral dose of ½ tablet of 500 mg sulfadoxine - 25 mg pyrimethamine per 10 kg of weigh: 1 tablet for weight less than 20 kg, 1.5 tablets for 20 to 29 kg, and 2 tablets for children who weigh 30 kg or more.

#### *Piperaquine*

PQ is not approved as a single entity. A fixed dose combination of PQ with dihydroartemisinin (DHA) was approved by the European Medicines Agency and marketed as Eurartesim for the treatment of uncomplicated malaria in children and infants of six months or older. The exact mechanism of action of PQ is unknown. PQ likely mirrors its closest structural analogue chloroquine. Chloroquine binds to toxic haeme (derived from the patient’s haemoglobin) within the parasite and prevents its detoxification. The Cmax and the AUC0-24 of PQ is 179 nm/ml and 1.679 ng/ml*h, respectively. The elimination half-life of PQ is around 20 days for paediatric patients. Due to its slow elimination, PQ may accumulate in plasma after multiple doses. In healthy volunteers, PQ exposure is increased approximately three-fold when administered with a high fat/high calorie meal. In combination curative treatment with DHA, PQ is safe and efficacious at 60 to 73.9 mg/kg dose in three daily doses against uncomplicated *P. falciparum* malaria [29,30]. However, because participants in this trial are healthy individuals, the parasite carriage is expected to be low. Taking account of the ED90 of PQ at 1.68 ± 0.210 mg/kg/day for *P. berghei* K-173, its CT100 of 42 hours and the long elimination half-life [31], PQ 320 mg tablets manufactured by Sigma Tau will be used at two treatment doses of 16 to 24 mg/kg body weight at 24-hour intervals (in combination with SP) as follows: 1 tablet for weigh 15 to 19 kg, 1.5 tablets for 20 to 29 kg, 2 tablets for 30 to 39 kg, and 2.5 tablets for 40 kg or more. The treatment will be administered with water under fasting conditions.

### Non-investigational products

#### *Albendazole*

Albendazole is a benzimidazole anthelmintic agent highly effective against a wide range of intestinal helminths and, at higher doses, against tissue helminth infections. Albendazole exhibits larvicidal, ovicidal and vermicidal activity, and is thought to act via inhibition of tubulin polymerization. This causes a cascade of metabolic disruption, including energy depletion, which immobilizes and then kills the susceptible helminth. Albendazole is poorly absorbed from the gastrointestinal tract due to its low aqueous solubility. Oral bioavailability appears to be enhanced when albendazole is co-administered with a fatty meal (estimated fat content 40 g) as evidenced by higher (up to five-fold on average) plasma concentrations of albendazole sulfoxide as compared to the fasted state. Plasma concentrations of albendazole sulfoxide increase in a dose-proportional manner over the therapeutic dose range following ingestion of a fatty meal (fat content 43.1 g). The mean apparent terminal elimination half-life of albendazole sulfoxide typically ranges from 8 to 12 hours in normal subjects. Albendazole sulfoxide is 70% bound to plasma protein and is widely distributed throughout the body. Following oral administration, albendazole has not been detected in human urine. Biliary elimination presumably accounts for a portion of the elimination [32].

Albendazole is used in children of 1 to 2 years at one oral 200 mg dose and in adults and children older than 2 years at 400 mg in one oral dose. Albendazole will be given at enrolment and four-month intervals in accordance with the WHO guideline [26].

#### *Praziquantel*

Praziquantel is a trematodicide used for oral treatment of infections due to schistosome and liver fluke. Praziquantel induces a rapid contraction of schistosomes by a specific effect on the permeability of the cell membrane. The drug further causes vacuolization and disintegration of the schistosome tegument. After oral administration praziquantel is rapidly absorbed (80%), subjected to a first pass effect, metabolized and eliminated by the kidneys. Maximal serum concentration is achieved one to three hours after dosing. The half-life of praziquantel in serum is 0.8 to 1.5 hours. However, in patients with moderate-to-severe hepatic dysfunction (Child-Pugh class B and C), praziquantel half-life, C max, and AUC increased progressively with the degree of hepatic impairment. In patients with schistosomiasis the half-life is 2.99 hour ± 1.28, the T max is 1.48 hour ± 0.74, the C max is 0.83 μg/mL ± 0.52 and the AUC is 3.02 μg/mL hour ± 0.59 (34). Praziquantel will be given at 40 mg/kg at enrolment and at the twelve-month follow-up [33].

### Concomitant treatments

The administration of paracetamol (acetaminophen) is allowed if the patient’s condition warrants it and should be recorded in the appropriate section of the CRF. Drugs with antimalarial activity (such as co-trimoxazole, macrolides, tetracycline or doxycycline) should be reported as concomitant medications. All concomitant medications taken by the patient during the study, from the date of signature of the informed consent are recorded in the appropriate section of the CRF.

### Safety assessment

Safety and tolerability of the treatments are evaluated by recording AEs and grading, laboratory, and vital signs evaluations [34]. All AEs are recorded and classified in accordance with their severity, seriousness, relationship and outcome. The severity of a clinical AE is scored as: mild when the patient is aware of the sign or symptom, but it is easily tolerated; moderate when the discomfort is enough to cause interference with usual activity; severe when the event causes incapacity with inability to work or perform usual activity; and life-threatening when the patient is at risk of death at the time of the event.

The investigators assess the drug-event relationship of each AE/SAE by using clinical judgment. Alternative causes, such as natural history of the underlying conditions or diseases, concomitant therapy, other risk factors and the temporal relationship of the event to the investigational product are considered and investigated. The investigator also consults the drug information and the Data and Safety Monitoring Board as needed in the determination of his/her assessment. For every AE, it is very important that the investigator always make an assessment of causality prior to transmission to the Trial Management Group even if the investigator disposes of minimal information to include in the initial report to the Trial Management Group. The investigator may change his/her opinion of causality in the light of follow-up information, amending the SAE case report form accordingly.

The outcome of each AE must be assessed according to the following classification: completely recovered, not yet completely recovered, deterioration, permanent damage, death, ongoing, or unknown.

All serious AEs, whether or not deemed drug-related, or expected, must be reported immediately or within 24 hours (one working day), using the Serious Adverse Event Notification Form by telefax or email to the sponsor. The report should state “Urgent Serious Adverse Event” and highlight the study code on the cover page. All other AEs not fulfilling the criteria of immediate reporting are recorded on the Case Report Form. This AE information is collected on a regular basis during the clinical trial.

### Statistical analysis plan

The effect of the type of intervention (either IPT versus the controls, or SP versus SP-P) will all be analysed using linear mixed model analysis. In each analysis, Hb concentration is modeled versus time, testing whether the change of Hb concentration over time differs between the two types of intervention. In each case, a linear mixed model is fitted with Hb concentration as outcome variable, and fixed effects including the type of intervention (indicator variable), time and the interaction between them. In these models, the effect of interest is the interaction between intervention and time, which formally tests the null hypothesis that the change in Hb concentration with time is the same in all interventions. Significance of the interaction term is tested using the F-test with a Kenwardroger correction for the number of degrees of freedom. As the study includes several (longitudinal) measurements within the same individual, and since the individuals cluster within schools, the dependency between individuals will be accounted for by including random effect terms into the model. More particularly, the model will include random effects (intercept and slope) for individual, nested within the random effect of school. An additional advantage of this technique is that it gives unbiased results in case individuals are lost to follow-up during the study.

Analysis of the data will be performed using the nlme package from the statistical software R or the proc mixed procedure from the software package SAS. All statistical analyses will be performed in close collaboration with StatUa, the Center for Statistics at the University of Antwerp.

### Medicines accountability procedures

The study site maintains the study drugs under adequate security. Study drugs sent to the study centre are verified by the investigator or designee; the amount sent and the exact supplies received are documented by signing and dating the appropriate shipping documents. Study drugs are dispensed as per the randomization list to each patient who meets the enrolment criteria. The investigators or designees record the patient’s number, patient’s initials and date dispensed on the Drug Accountability Form. The investigators maintain an accurate running accountability of the study drugs. At the end of the study, final disposal of the unused drugs will be decided by the sponsor after final reconciliation has been made.

### Case report form

All data and observations are documented and entered in the CRF. For this study, the CRF allows identification of the study site and patient; recording of the selection and inclusion of patients in the study; recording of all data collected at each different study visit; recording of possible AE and any suspension of the study. Participants are identified by their initials and a study identification number on the CRF. When recording data on the CRF and entering the data in the database, confidentiality and security of the forms are ensured at all steps. All corrections are made on CRFs by striking through the incorrect entry with a single line and entering the correct information adjacent to it. The correction is initialed and dated by the investigator. Additional records are kept in the clinical and laboratory record books at the core facility in Kinshasa. The investigators verify data at each study visit.

### Randomisation and treatment allocation

Children fulfilling the inclusion/exclusion criteria will be assigned a sequential study number and will be treated according to a predetermined randomization list of blocks of varying size per school. Within each block each schoolchild will randomly be assigned to one of the two intervention groups or the control group in 1:1:1 balance. Sealed envelopes labeled with the school unique code and containing the list with the treatment allocated to the participants will be provided according to the above mentioned list and opened at the moment the children are recruited.

### Treatment administration

The study treatment is administered under the direct supervision of a study nurse. Study drugs are given to the children consistent with the investigational brochure of each product. After drug administration, children will be kept for thirty minutes in the class. In case vomiting occurs within 30 minutes of administration a dose is repeated in full. This event is documented in the CRF. If vomiting persists beyond two additional doses, the child is withdrawn from the study.

Upon arrival at a school, an inventory is performed and a drug receipt log filled out and signed by the staff. Accountability for the drug at all school sites is the responsibility of the principal investigator. The investigators ensure that the drug is used only in accordance with this protocol. Accountability records include dates, quantities, lot numbers, expiration dates, and patient numbers.

### Quality assurance

To ensure the rights, safety and well-being of the human subjects enrolled in this trial and to validate the Investigator’s adherence to the protocol, all applicable regulations of the International Conference on Harmonisation (ICH) and the Good Clinical Practices (GCP) guidelines are strictly applied. The quality assurance of records and data are guaranteed. The study clinicians are trained about the study protocol prior to the onset of the trial. The study clinicians complete case records at each hospital visit. The medical records of the clinicians are transferred to a CRF by the investigators. At school visits, the investigators record data on the CRF. The investigators carry out 100% Source Data Verification on the data collected at the regional hospital centre during the passive follow-up to guarantee the best conduct of the study. To ensure accuracy of the data, a CRF completed by an investigator is contra-checked by another investigator. The consistence of data of each participant is checked by the investigators at each follow-up visit by cross-checking the reports of the school teachers and of parents/guardians, and the medical records at the regional hospital centre. The investigators, therefore, have frequent contacts with the clinicians and the school teachers.

### Data management

All data is processed from the CRF into an electronic database by two different persons after each follow-up visit. Then the two files are merged. Unmerging information is verified and corrected and added to the final database. During the conduct of the study, data are verified and reviewed to produce and maintain high quality data. All unresolved issues are queried and resolved before locking the database. Data transfer and handling are done with appropriate security measures and with regard to rights, safety and well-being of trial subjects. Two back-up files of the database are stored on compact discs after each data entry session. For quality control, check programs are written into the database to limit the entry of incorrect data and ensure entry of data into required fields.

### Ethical considerations

The Investigators agreed to conduct the present study in full agreement with the principles of the 'Declaration of Helsinki’ and subsequent relevant amendments. All research activities will be conducted in accordance with the standards and codes of conduct accepted by the ICH guidelines. The study is approved by the Ethical Committees of the University of Antwerp, Belgium and of the School of Public Health, University of Kinshasa, DRCongo. Approval was also obtained from the Ministry of Health of DRCongo. Prior to the start of the project, special permission was obtained from the DRCongo Ministry of Education and the local health and education authorities of Biyela. Written informed consent was obtained from the guardians for all children before entering the study. An assent was obtained, as well, from children who were 12-years-old or older. In the control arm no treatment is given as no foreseeable serious risk can be anticipated and no standard of care yet exists in schoolchildren. All participants are followed-up, examined and treated free of charge if malaria or other illnesses occur during the period of the study.

To measure Hb level and *P. falciparum* parasitaemia, finger pricks are done at each visit for hemocontrol and microscopy. A total of 3 ml of venous blood is collected at baseline and month 12 for genotyping and other tests that the clinician may request for the management of illnesses. Residual plasma is separated, stored and transported at -70°C to be assayed by ELISA and fluorescence activated cell sorting (FACS) for malarial cytokines and antibody quantification and other malaria-related parameters (described in an independent protocol after the operational part of the study is finished). The human genome will not be examined.

## Discussion

Little research has been performed on IPTsc in malaria stable transmission. Rohner *et al.*[35] found no effect for IPT with SP monotherapy given at baseline and three months later to schoolchildren. On the other hand, Clarke *et al*. [27] found a reduced prevalence of anaemia by 50% for a 'sulfadoxine-pyrimethamine plus amodiaquine’ IPT combination therapy given three times at four-month intervals at one year follow-up compared to a placebo. They also reported a significant improvement from the IPT intervention on the behavioural and educational achievements of the schoolchildren. Similarly, Nankabirwa *et al*. [36] reported a decreased risk of malaria parasitaemia following 42 days follow-up for dihydroartemisinin-piperaquine (11.7% (95%CI:7.9 to 17.1)) and 'amodiaquine plus SP’ (44.3% (95%CI:37.6 to 51.5)) compared to a SP monotherapy (79.7%(95% CI:73.6 to 85.2)), and to a placebo (84.6%(95% CI: 79.1 to 89.3)).

Our clinical trial is unique in investigating the impact of antimalaria IPT in schoolchildren of hyper endemic areas for longer follow-up, resistance markers for SP at baseline and after 12 months (including break through parasitaemias). Our study is also unique in using piperaquine in combination with SP. Artemisinin derivative has been excluded as it is part of the treatment policies in virtually all malaria endemic countries [37].

The findings of the clinical trial are, therefore, expected to make a major contribution to the public health of youngsters who fail to thrive and grow due to multiple morbidities.

## Trial status

Recruiting.

### Informed consent

Written informed consent was obtained from the participants’ parents or guardians for the conduct of the study and the publication of this manuscript. As part of the clinical trial protocol, an information sheet for participants and an informed consent form were reviewed and approved by the Ethical Committee of the University of Antwerp, Belgium, the Ethical Committee of the School of Public Health, University of Kinshasa, DRCongo and the Ministry of Health of DRCongo. A copy of the information sheet and the written consent form in French, English and in the local language are available for review by the Editor-in-Chief of the Trials Journal.

## Abbreviations

AE: Adverse events; CRF: Case report form; DHA: Dihydroartemisinin; DHFR: Dihydrofolate reductase; DHPS: Dihydropteroate synthase; ELISA: Enzyme-linked immunosorbent assay; FACS: Fluorescence activated cell sorting; GCP: Good Clinical Practice; H/A: Height for age; ICH: International Conference on Harmonisation; IPT: Intermittent preventive treatment; IPTc: Intermittent preventive treatment in children; IPTi: Intermittent preventive treatment in infancy; IPTp: Intermittent preventive treatment in pregnancy; IPTsc: Intermittent preventive treatment in schoolchildren; LIC: Low income countries; MIC: Minimal inhibitory concentration; PCR: Polymerase chain reaction; PQ: Piperaquine; RCT: Randomised controlled trial; RR: Relative risk; SAE: Serious adverse events; SP: Sulfadoxine-pyrimethamine; SP-PQ: Sulfadoxine-pyrimethamine plus piperaquine; SSA: Sub Saharan Africa; STH: Soil transmitted helminths; W/H: Weight for height.

## Competing interests

The research is funded by the Flemish Interuniversity Council (VLIR-UOS) and the Research Foundation - Flanders (FWO) of Belgium. The authors declare that they have no competing interests.

## Authors’ contributions

JYD and JPVg conceptualised the idea. JYD wrote the study protocol and the trial essential documents. JM contributed to the essential documents writing. JYD and JM did the approval procedures of the protocol by ethical committees and regulatory authorities. JYD and JM implement the study protocol in the field. PL and JPVg reviewed the protocol and supervise the conduct of the trial. JPVg carries the sponsorship of the study. All authors read and approved the submitted manuscript.

## Authors’ information

JYD is a Medical Doctor, epidemiologist and drug regulatory consultant. He is affiliated to the International Health Unit, Department of Epidemiology and Social Medicine, Faculty of Medicine and Health Sciences, University of Antwerp. JM is a Medical Doctor, currently MSc student. He is affiliated to the Parasitology Department, Faculty of Medicine, University of Kinshasa. PL is a Medical Doctor, epidemiologist, PhD. He is professor at the Parasitology Department, Faculty of Medicine, University of Kinshasa. JPVg is a Medical Doctor, epidemiologist, PhD. He has been involved for a decade in RCTs in Low Income Countries. He is heading the International Health Unit, Department of Epidemiology and Social Medicine, Faculty of Medicine and Health Sciences, University of Antwerp.

## Supplementary Material

Additional file 1 WHO criteria for malaria diagnosis (WHO 2010). Click here for file

Additional file 2 Patient flow at school visit. Click here for file

Additional file 3 Patient flow at hospital visit. Click here for file
