# Supplementary material for: Intermittent preventive treatment: efficacy and safety of sulfadoxine-pyrimethamine and sulfadoxine-pyrimethamine plus piperaquine regimens in schoolchildren of the Democratic Republic of Congo: a study protocol for a randomized controlled trial
Source: Trials. 2013 Sep 24;14:311. doi: 10.1186/1745-6215-14-311 (PMC4015766; doi:10.1186/1745-6215-14-311)
Supplement: Additional file 1 — WHO criteria for malaria diagnosis (WHO 2010). [file 1745-6215-14-311-S1.doc]

**ANNEX 1: W.H.**O CRITERIA FOR MALARIA DIAGNOSIS (WHO 2010)

| **Uncomplicated malaria**  Uncomplicated malaria is defined as symptomatic malaria without signs of severity or evidence (clinical or laboratory) of vital organ dysfunction. The signs and symptoms of uncomplicated malaria are nonspecific. Malaria is, therefore, suspected clinically mostly on the basis of fever or a history of fever.   - Fever or a history of fever (other possible causes of fever must be carefully considered). in settings where the risk of malaria is low, clinical diagnosis of uncomplicated malaria should be based on the possibility of exposure to malaria and a history of fever in the previous three days with no features of other severe diseases; In settings where the risk of malaria is high, clinical diagnosis should be based on a history of fever in the previous 24 h and/or the presence of anaemia, for which pallor of the palms appears to be the most reliable sign in young children. - Clinical suspicion of malaria should be confirmed with a parasitological diagnosis In all settings. However, in settings where parasitological diagnosis is not possible, the decision to provide antimalarial treatment must be based on the prior probability of the illness being malaria. Other possible causes of fever and need for alternative treatment must always be carefully considered.   **Severe malaria**  In a patient with *P. falciparum* asexual parasitaemia and no other obvious cause of symptoms, the presence of one or more of the following clinical or laboratory features classifies the patient as suffering from severe malaria.   - Clinical features: - impaired consciousness or unrousable coma - prostration, i.e. generalized weakness so that the patient is unable walk - or sit up without assistance - failure to feed - multiple convulsions – more than two episodes in 24 h - deep breathing, respiratory distress (acidotic breathing) - circulatory collapse or shock, systolic blood pressure < 70 mm Hg in adults - and < 50 mm Hg in children - clinical jaundice plus evidence of other vital organ dysfunction - haemoglobinuria - abnormalspontaneousbleeding - pulmonaryoedema (radiological) - Danger Signs - recent convulsions (>1 within 24 h) - altered consciousness (confusion) - lethargy - unable to drink or breast feed - vomiting everything - unable to stand/sit due to weakness - Laboratory findings: - hypoglycaemia (blood glucose < 2.2 mmol/l or < 40 mg/dl) - metabolic acidosis (plasma bicarbonate < 15 mmol/l) - severe normocytic anaemia (Hb < 5 g/dl, packed cell volume < 15%) - haemoglobinuria - hyperparasitaemia (> 2%/100 000/μl in low intensity transmission areas or > 5% - or 250 000/μl in areas of high stable malaria transmission intensity) - hyperlactataemia (lactate > 5 mmol/l) - renal impairment (serum creatinine> 265 μmol/l). |
| --- |
