# Supplementary material for: Intermittent preventive treatment: efficacy and safety of sulfadoxine-pyrimethamine and sulfadoxine-pyrimethamine plus piperaquine regimens in schoolchildren of the Democratic Republic of Congo: a study protocol for a randomized controlled trial
Source: Trials. 2013 Sep 24;14:311. doi: 10.1186/1745-6215-14-311 (PMC4015766; doi:10.1186/1745-6215-14-311)
Supplement: Additional file 2 — Patient flow at school visit. [file 1745-6215-14-311-S2.doc]

**ANNEX 2: PATIENT FLOW AT SCHOOL VISIT**

YES

YES

YES

YES

NO
ESchool

NO
ESchool

Mass information parents of 1 – 5 schoolclass

Collect the samples (1 for stool and 1 for urines) that were taken at home

Do the informed consenting

Informed consent signed and ascent (if =<12 years)

Social patters, anthropometric parameters, temperature, cursory physical examination

Prick or venous blood sample collection

Confection of blades

Reading of the blades

Treatment allocation and treatment administration

NO

NO

- Weight < 14 kg
- Participant to a trial in previous 4 weeks
- Hypersensitivity or SAE
- Clinical malaria symptoms
- Fever
- Decompasated anaemia
- Serious chronic diseases
- Body weight < 14 kg
- HIV tuberculosis

Exclude

Exclude
