# Supplementary material for: Intermittent preventive treatment: efficacy and safety of sulfadoxine-pyrimethamine and sulfadoxine-pyrimethamine plus piperaquine regimens in schoolchildren of the Democratic Republic of Congo: a study protocol for a randomized controlled trial
Source: Trials. 2013 Sep 24;14:311. doi: 10.1186/1745-6215-14-311 (PMC4015766; doi:10.1186/1745-6215-14-311)
Supplement: Additional file 3 — Patient flow at hospital visit. [file 1745-6215-14-311-S3.doc]

**ANNEX 3 : PATIENT FLOW AT HOSPITAL VISIT**

Record collected data on the CRF

Samples collection and staining of blades (blood smear, stool and urines)

If an enrolled schoolchild is sick

Take the child health visit card

Bring the child to the Biyela regional hospital

Contact the parents and inform CS

Anthropometric measurements and vital signs

Physical examination + Lab

Treatment and hospitalisation (if required)

Take the child health visit card

Bring the child to Biyela regional hospital

Contact the parents and inform CS

Report SAEs and exclude patient from the study

At home

At school
